# Knowledge and Response to Stroke Among Lebanese Adults: A Population-Based Survey

**DOI:** 10.3389/fpubh.2022.891073

**Published:** 2022-06-03

**Authors:** Sylvia Saade, Souheil Hallit, Pascale Salameh, Hassan Hosseini

**Affiliations:** ^1^Life Sciences and Health Department, Paris-Est University, Paris, France; ^2^Health and Sciences Department, American University of Health and Sciences, Beirut, Lebanon; ^3^School of Medicine and Medical Sciences, Holy Spirit University of Kaslik, Jounieh, Lebanon; ^4^Psychology Department, College of Humanities, Effat University, Jeddah, Saudi Arabia; ^5^Research Department, Psychiatric Hospital of the Cross, Jal El Dib, Lebanon; ^6^INSPECT-LB: Institut National de Santé Publique, Épidémiologie Clinique et Toxicologie-Liban, Beirut, Lebanon; ^7^Faculty of Public Health, Lebanese University, Beirut, Lebanon; ^8^Faculty of Medicine, University of Nicosia, Nicosia, Cyprus; ^9^Faculty of Pharmacy, Lebanese University, Beirut, Lebanon

**Keywords:** stroke, awareness, knowledge, practice, Lebanon

## Abstract

**Objectives:**

To date, research on public awareness of stroke warning symptoms, risk factors and practice in the general adult population in Lebanon is scarce. The aim of our study is to identify the level of stroke awareness in order to develop and implement preventive measures particularly in relationship to primary stroke prevention.

**Methods:**

It is a cross-sectional study conducted among 410 adult participants from the five main governorates of Lebanon. Stroke knowledge and practice were assessed using two validated questionnaires namely the Stroke Knowledge Test (SKT) and the Stroke Action Test (STAT). Multivariable linear regression models were conducted to examine socio-demographic, social habits, and clinical factors independently associated with the SKT and the STAT scores.

**Results:**

The mean SKT score of the participants was 9.16. 48.5% showed a poor stroke-related knowledge level and 51.5% a good knowledge level. Living in Mount Lebanon and occasional smokers showed statistically significant lower mean SKT scores; whereas, university degree and suffering from diabetes mellitus were associated with higher mean SKT scores. The mean overall STAT score was 41.3%. For 36.8% of the stroke symptoms, respondents selected call 112. The mean STAT scores of participants who get their information from the internet was statistically significantly lower. However, no association was found between the SKT score and the STAT score.

**Conclusion:**

Knowledge of stroke risk factors was low, as was awareness of the need to call 112 in response to stroke symptoms. Hence, it is essential to develop health education programs in order to decrease stroke morbidity and mortality.

## Introduction

Globally, stroke is a primary cause of mortality and morbidity that results in high disability rates (1–4). Age, positive family history, chronic conditions such as diabetes, hypertension, and cardiovascular diseases are common risk factors of stroke. In addition, certain lifestyle habits including smoking and alcohol intake are also known stroke risk factors (5, 6). It is well-documented that 80% of stroke cases can be prevented through lifestyle changes and control of modifiable risk factors (3, 4), hence emphasizing the importance of primary prevention.

Over the years, the burden of stroke increased, its incidence has escalated in low and middle-income countries; while it declined in high-income countries to 42% (7–9). Regarding the Middle East, among the developing countries, the World Bank reported that stroke incidence rates have significantly increased over the last decade; they ranged from 22.7 to 250 per 100 000 per year in 2000–2014 compared to the gross rates of 112–223 per 100 000 per year in 2000–2008 among the high-income countries (10). Lebanon, a small country located in the Middle East on the eastern shore of the Mediterranean Sea, lacks data on stroke prevalence. According to the WHO, stroke is the second leading cause of mortality in Lebanon with 9.4% of all deaths in 2012. Furthermore, a study conducted in 2015 showed an adjusted prevalence of stroke of 0.5% and a world standardized prevalence of 0.6% in Lebanon which is higher than other emerging countries in the Middle East (11). It is noteworthy to mention that the prevalence of risk factors of stroke such as smoking, obesity, low physical activity, and hypertension is high in the Lebanese community (12, 13). In addition, Farah *etal*. reported that 1 out of 8 Lebanese residents without prior stroke or TIA experienced stroke symptoms and a positive association between stroke risk factors and stroke symptoms prevalence was seen (14). According to the World Economic Forum, Lebanon is ranked globally as the 10th best overall for quality education with a literacy rate of 93.9% (15). In 2012, a net enrolment rate of 93.3% was recorded in primary education and 46.3% in higher education (16). This is attributed to the limited availability of public higher education, with only one public university in the country. Nevertheless, because of the current economic and financial crisis in Lebanon, the rising tuition fees in private schools and universities is expected to negatively affect the affordability of education to low-income households.

Early hospitalization and timely treatment could prevent the high morbidity and mortality in stroke (17–19) but delay often occurs due to lack of knowledge about warning signs of stroke, poor infrastructure, and indecision about hospital admission despite the availability of appropriate infrastructure and facilities (20). Moreover, there is a lack of awareness that timely treatment of comorbidities and proper lifestyle modifications may reduce stroke occurrence as well as morbidity among survivors of stroke (21). Thus, preventative measures are warranted particularly regarding primary stroke prevention.

Worldwide, former studies have shown poor knowledge of stroke symptoms and risk factors in the general public (22–33). According to studies conducted in Jordan (34), in Uganda (35), and in Egypt (29), 47, 75, and 68.2% of the subjects couldn't identify any stroke risk factor, respectively. Moreover, Alreshidi et al. revealed that the majority of the Saudi population showed poor stroke risk factors and warning signs knowledge leading to insufficient KAP in 63.8% of the participants (36). Consequently, in primary stroke prevention, awareness is the first approach to diminish stroke incidence in the general public (37–41). Knowledge of stroke risk factors and warning signs is necessary in stroke prevention as well as the readiness in implementing prevention measures (37–42).

To date, research on public awareness of stroke warning symptoms, risk factors and practice in the general adult population in Lebanon is scarce. Therefore, a community-based survey aimed to evaluate the knowledge and response of Lebanese adults toward stroke is of outmost importance in order to identify the level of stroke awareness in order to develop and implement preventive measures particularly in relationship to primary stroke prevention.

## Methods

### Study Design and Sampling

A total of 410 participants were recruited randomly from the five main governorates of Lebanon between the months of February and August 2020. These include Beirut, Mount Lebanon, North, South and Bekaa. The regions of Mount Lebanon and Beirut share the highest number of inhabitants- around 75%- according to national registries.

In this cross-sectional study, participants were first given a brief explanation of the study, and consent was obtained upon filling a self-administered questionnaire. Due to the COVID-19 situation, it was modified to a google form survey in order to be able to proceed with data collection. According to the Epi info sample size calculations, providing a confidence level of 95%, a margin of error of 5%, and assuming 36% of the population have adequate KAP according to a study conducted in Saudi-Arabia (36), a total sample of 354 Lebanese adults were targeted.

Lebanese adults aged 18 years and over were included in the study. Participants under 18 or who do not hold a Lebanese nationality were excluded.

Review board approval was acquired on the 5th of October 2019 from the Lebanese University.

### Questionnaire

The instrument used was adopted from two validated and reliable stroke questionnaires namely the Stroke Knowledge Test (SKT) that has a validated Arabic version and the Stroke Action Test (STAT) which was translated into Arabic. The SKT and the STAT aimed at evaluating the participant's knowledge and practice toward stroke, respectively. The survey instrument was then piloted on 10 subjects prior to its finalization and distribution. The questionnaire's final version included 3 sections.

The first section was concerned with the respondents' socio-demographic characteristics, social factors and habits, family history, and medical profile. Registered data included age, gender, marital status, education level, social security insurance, occupation; whether they have ever known anyone who has ever had a stroke, where they get their health information. Presence of known stroke risk factors such as alcohol consumption, smoking, dyslipidemia, diabetes, hypertension, abnormal heart rhythm, previous cerebrovascular disease, previous MI, family history of cardiovascular or cerebrovascular disease.

The second part investigated the knowledge of stroke, risk factors and warning signs of stroke using the SKT.

The SKT is a 20-items questionnaire developed by Sullivan and Dunton (43). The cut off point for good knowledge level is 50% of the 20 items. The tool has demonstrated good internal consistency, test-retest reliability and construct validity (25, 43).

Results from psychometric investigations were established at Cronbach of 0.65 in the original questionnaire demonstrating its reliability and validity in stroke knowledge assessment (43). The instrument is used worldwide and has been translated into other languages including Arabic by Eshah (34) in her study entitled “Knowledge of Stroke and Cerebrovascular Risk Factors in Jordanian adults”. For the Arabic version, face and content validity were established with a Cronbach's alpha of 0.68 (34). A copy of the tool in both versions with the answers were obtained by directly contacting Dr. Sullivan and Pr. Eshah through email and permission to use the tool was granted. Minor adjustments were made to the Arabic version by changing certain terminologies as dialects differ in different Arabic countries (44).

Reliability of the instrument in our study was established with a Cronbach alpha of 0.877.

The last part of the survey explored appropriate practice toward stroke using the STAT. It is a 21 items questionnaire developed by Billings-Gagliardi and Mazor (45) in the study entitled “Development and Validation of the Stroke Action Test.” The objective of the study was to develop and assess a tool where the participant needs to link individual stroke and non-stroke symptoms with the most appropriate action. The reliability of the 28-item test was good as determined by a Cronbach's alpha of 0.83 (45).

A copy of the STAT tool was obtained by contacting Dr. Billings-Gagliardi directly by email and permission to use the tool granted. The instrument was translated to Arabic by two bilingual experts and back translation into English was performed by two additional bilingual experts. The language experts compared the original English instrument with the back translated Arabic instrument and edited to obtain the matched Arabic version.

Reliability of the instrument in our study was established with a Cronbach alpha of 0.923.

### Data Analysis

Statistical analyses were performed using Statistical Package for Social Science (SPSS) version 23 (IBM SPSS Software, Chicago, IL, USA). Descriptive statistics were calculated using mean and standard deviation for continuous measures, counts and percentages for categorical variables.

A reliability test was performed for each of the questionnaires used using Cronbach's alpha to determine internal consistency. Data was weighted according to age and gender. The assumptions of the statistical tests were checked. The Student *t*-test and ANOVA test were used to assess the association between each continuous independent variable (knowledge: SKT score, and practice scores) and the sociodemographic variables. Multivariable linear regression models were conducted to examine factors associated with the SKT and the STAT scores as dependent variables and taking all variables that showed a *p* < 0.25 (46) in the bivariate analysis as independent variables. A two-sided *p* < 0.05 was used to indicate statistical significance.

## Results

### Socio-Demographic Characteristics and Clinical Profile

A total of 410 participants from the 5 districts of Lebanon completed the questionnaire. The respondents' socio-demographic characteristics, social factors and habits, family history and medical profile are summarized in Table 1 in Appendix A.

The study population included 281 women (68.5%) and 129 males (31.5%), with 77.6% aged between 18 and 44 and 22.4% aged 45 and above. The results showed that 48% of the subjects were married, 37.3% without any professional activity, 62% with social security insurance and 89% had completed a university degree. In addition, the majority of participants (48%) lived in Mount Lebanon and 20.2% in Beirut. 23.7 and 55.9% of the respondents were occasional smokers and alcohol consumers, respectively. Around 45% of the participants had a family member or a friend who suffered a stroke.

Of the 410 respondents, many modifiable stroke risk factors were observed. The most prevalent were diabetes (89%) and dyslipidemia (86.9%) followed by hypertension (63.8%) and atrial fibrillation (49.4%). Moreover, 29.7% declared getting their health information from television and 22.6% from a health or fitness center.

### Knowledge of Stroke

The mean SKT score of the participants was 9.16. Respondents were divided into 2 groups based on their SKT scores. 48.5% (*n* = 199) scored <50% and therefore showed a poor stroke-related knowledge level and 51.5% (211) a good knowledge level.

In addition, 61% of the sample was able to identify the pathological mechanism of stroke.

Moreover, of the 8 modifiable risk factors considered, the average score for knowledge was 3.49 of 8. 12.4% of participants were not able to identify 1 risk factor, around 20% were able to recognize 4 and approximately 43% were unable to enumerate more than 3. The most frequently recognized risk factors were physical inactivity (70.5%), obesity (69.3%), and hyperlipidemia (67.3%) followed by hypertension (52.9%).

In the bivariate analysis, being between 18 and 44 years old (*p* = 0.018), having a university degree compared with secondary education (*p* < 0.001), social security insurance (*p* = 0.02) and a medical history of diabetes mellitus (*p* = 0.07) were significantly associated with a higher SKT score. On the other hand, the mean SKT scores of participants living in Mount Lebanon compared with Beirut (*p* < 0.0001), who are occasional smokers vs. non-smokers (*p* = 0.015), occasional alcohol drinkers compared with non-drinkers (*p* = 0.035), who have a medical history of hyperlipidemia (*p* = 0.007) and who get their health information form the internet (*p* = 0.016) were significantly lower (Table 1).

**Table 1:** Bivariate analysis of factors associated with the SKT.

**Social and demographic data**	
**Variable**	**Mean** **±SD**
**Gender**	
Male	48.92 ± 23.476
Female	46.60 ± 23.538
*p*-value	0.32
**Age**	
18-44	49.86 ± 22.286
45 and above	43.87 ± 25.281
*p*-value	0.018
**Patient's marital status**	
Single	49.31 ± 22.685
Married	46.88 ± 24.247
Divorced	42.87 ± 21.916
*p*-value	0.437
**Patient's level of education**	
Primary/complementary education (Elementary)	50.91 ± 31.363
Secondary education (high school)	63.07 ± 20.232
Universal education	45.81 ± 22.988
*p*-value	<0.001
**Work status**	
Person without any professional activity (I don't work)	48.22 ± 21.532
Worker	48.38 ± 23.653
Unemployed	43.14 ± 25.439
Retired	47.17 ± 24.828
*p*-value	0.660
**Does the patient have social security insurance?**	
No	43.42 ± 25.326
Yes	50.848 ± 21.684
*p*-value	0.002
**Region/Governorate**	
Beirut	44.87 ± 25.157
Mount Lebanon	42.82 ± 22.812
Other	52.89 ± 22.611
*p*-value	<0.0001
**Current smoking**	
Every day	43.91 ± 24.896
Occasionally	53.62 ± 21.402
Never	46.99 ± 23.424
*p*-value	0.015
**Have you ever smoked at least 100 cigarettes throughout your life? [5 packages** **=** **100 cigarettes]**	
Yes	44.50 ± 23.528
No	48.96 ± 23.426
*p*-value	0.091
**Daily alcohol consumption**	
Occasionally	45.88 ± 23.088
Never	50.81 ± 23.595
*p*-value	0.035
**Has any family member or close friend suffered a stroke?**	
No	47.45 ± 23.792
Yes	48.24 ± 23.194
*p*-value	0.736
**Family history of Heart failure**	
No	47.27 ± 23.447
Yes	51.49 ± 23.677
Don't know	47.76 ± 23.893
*p*-value	0.530
**Family history of Stroke**	
No	46.80 ± 23.829
Yes	49.35 ± 22.042
Don't know	55.54 ± 21.208
*p*-value	0.112
**Family history of myocardial infarction**	
No	47.32 ± 24.027
Yes	47.60 ± 23.924
Don't know	51.26 ± 18.448
*p*-value	0.633
**Family history of atrial fibrillation**	
No	45.63 ± 23.768
Yes	50.47 ± 23.701
Don't know	48.73 ± 22.047
*p*-value	0.165
**Have you ever had/Do you have Stroke? If yes, was this in the last 12 months?**	
No	47.72 ± 23.553
Yes	50.19 ± 22.721
*p*-value	0.718
**Have you ever had/Do you have Heart Attack ? If yes, was this in the last 12 months?**	
No	47.32 ± 23.462
Yes	53.71 ± 23.639
*p*-value	0.149
**Have you ever had/Do you have Angina? If yes, was this in the last 12 months?**	
No	47.95 ± 23.462
Yes, in the last 12 months	44.88 ± 24.775
*p*-value	0.567
**Have you ever had/Do you have transient ischaemic attack (TIA)? If yes, was this in the last 12 months?**	
No	47.55 ± 23.589
Yes	57.95 ± 17.912
*p*-value	0.171
**Have you ever had/Do you have diabetes? If yes, was this in the last 12 months?**	
No	47.29 ± 23.592
Yes, in the last 12 months	39.55 ± 24.533
Yes, long ago	59.55 ± 17.719
*p*-value	0.007
**Have you ever been told by a doctor that you have high blood pressure?**	
No	47.57 ± 23.286
Yes, but without medication	49.39 ± 23.553
Yes, with medication	48.66 ± 25.309
*p*-value	0.923
**Have you ever been told by a doctor that you have high cholesterol?**	
No	49.65 ± 22.577
Yes, but without medication	41.11 ± 27.783
Yes, with medication	39.94 ± 23.593
*p*-value	0.007
**Have you ever been told by a doctor that you have abnormal heart rhythm (atrial fibrillation)?**	
No	48.27 ± 23.330
Yes, but without medication	46.24 ± 27.607
Yes, with medication	41.11 ± 23.365
*p*-value	0.400
**Where do you get most of your health information? Internet**	
No	51.80 ± 22.803
Yes	45.85 ± 23.636
*p*-value	0.016
**Where do you get most of your health information? Newspaper/Magazine**	
No	47.04 ± 23.957
Yes	52.81 ± 19.724
*p*-value	0.056
**Where do you get most of your health information? Television**	
No	46.42 ± 23.393
Yes	51.53 ± 23.513
*p*-value	0.051
**Where do you get most of your health information? Health/fitness center**	
No	47.93 ± 23.391
Yes	46.22 ± 25.086
*p*-value	0.686
**Where do you get most of your health information? Friends**	
No	47.43 ± 23.462
Yes	49.06 ± 23.742
*p*-value	0.556
**Where do you get most of your health information? Church**	
Yes	47.70 ± 23.544
No	58.69 ± 17.925
*p*-value	0.374
**Where do you get most of your health information? Alternative practitioners**	
No	47.52 ± 23.536
Yes	55.92 ± 21.878
*p*-value	0.201
**Where do you get most of your health information? Nurse**	
No	47.50 ± 23.687
Yes	50.25 ± 22.056
*p*-value	0.463
**Where do you get most of your health information? Doctor**	
No	47.15 ± 23.969
Yes	48.30 ± 23.176
*p*-value	0.625
**Where do you get most of your health information? Family member**	
No	47.57 ± 23.298
Yes	48.75 ± 24.512
*p*-value	0.693
**Where do you get most of your health information? Don't know**	
No	47.90 ± 23.681
Yes	46.57 ± 21.699
*p*-value	0.757

In the multivariate linear regression, living in Mount Lebanon compared with Beirut (ß = −8.819, *p* < 0.001) and occasional smokers compared with non-smokers (ß = −13.099, *p* = 0.016) showed statistically significant lower mean SKT scores; whereas, university degree vs. secondary education (ß = 7.217, *p* = 0.006) and suffering from diabetes mellitus (ß = 12.719, *p* = 0.01) were associated with higher mean SKT scores (Table 2).

**Table 2:** Multivariate analysis taking the SKT as the dependent variable.

**Linear regression taking the SKT as the dependent variable**					
**Variable**	**Unstandardized beta**	**Standardized beta**	***p*** **-value**	**Confidence interval**	
Region-ML/Beirut^*^	−8.819	−0.190	<0.001	−13.195	−4.443
Education-secondary/University^*^	7.217	0.133	0.006	2.091	12.343
Current smoking: occasionally/No smoking^*^	−13.099	−0.116	0.016	−23.708	−2.489
Medical history of diabetes-yes long ago/None^*^	12.719	0.124	0.010	3.045	22.393

* *Reference group*.

### Response to Stroke

The mean overall STAT score was 41.3%. On average, for 36.8% of the stroke symptoms, respondents selected call 112. Moreover, of the 21 stroke symptoms considered, ~41.5% of respondents were unable to identify the most appropriate action to more than 6 stroke symptoms and around 14% responded correctly to all symptoms.

The stroke symptoms for which the fewest percentage of respondents would call 112 were “sudden loss of coordination” (0.7%), followed by “sudden confusion” (24.6%), “sudden dizziness” (25.1%) and “sudden severe headache with no known cause” (28%). On the other hand, call 112 was greatly chosen by the participants in situations where difficulty speaking presented together with sudden arm and face weakness. Moreover, 82.4% of the sample recognized the need to call 112 when the diagnosis of stroke was provided.

In the bivariate analysis, medical history of heart attack and atrial fibrillation were significantly associated with a higher mean STAT score (*p* = 0.026 and *p* = 0.002, respectively). On the other hand, getting health information from the internet was significantly linked to a lower mean STAT score (*p* < 0.001; Table 3).

**Table 3:** Bivariate analysis of factors associated with STAT score.

**Social and demographic data**	
**Variable**	**Mean** **±SD**
**Gender**	
Male	43.64 ± 26.550
Female	41.15 ± 23.531
*p*-value	0.316
**Age**	
18–44	42.44 ± 24.577
45 and above	42.19 ± 25.977
*p*-value	0.924
**Patient's marital status**	
Single	43.74 ± 24.026
Married	40.86 ± 26.005
Divorced	48.67 ± 21.198
*p*-value	0.335
**Patient's level of education**	
Primary/complementary education (Elementary)	57.33 ± 21.405
Secondary education (high school)	39.10 ± 30.207
University education	42.17 ± 24.324
*p*-value	0.096
**Work status**	
Person without any professional activity (I don't work)	40.62 ± 24.547
Worker	43.88 ± 25.422
Unemployed	39.33 ± 22.098
Retired	37.40 ± 25.771
*p*-value	0.361
**Does the patient have social security insurance?**	
No	41.65 ± 24.648
Yes	43.37 ± 25.625
*p*-value	0.492
**Region/Governorate**	
Beirut	38.94 ± 24.048
Mount Lebanon	43.28 ± 23.776
Other	42.62 ± 26.378
*p*-value	0.514
**Current smoking**	
Every day	41.85 ± 26.916
Occasionally	43.59 ± 24.453
Never	42.06 ± 24.605
*p*-value	0.865
**Have you ever smoked at least 100 cigarettes throughout your life? [5 packages** **=** **100 cigarettes]**	
Yes	44.79 ± 27.637
No	41.49 ± 24.040
*p*-value	0.275
**Daily alcohol consumption**	
Occasionally	42.48 ± 25.973
Never	42.33 ± 23.749
*p*-value	0.954
**Has any family member or close friend suffered a stroke?**	
No	41.03 ± 24.058
Yes	43.39 ± 25.783
*p*-value	0.345
**Family history of heart failure**	
No	41.76 ± 24.997
Yes	48.51 ± 26.259
Don't know	40.59 ± 23.861
*p*-value	0.207
**Family history of stroke**	
No	42.97 ± 25.554
Yes	38.94 ± 20.537
Don't know	41.04 ± 25.866
*p*-value	0.559
**Family history of myocardial infarction**	
No	42.80 ± 26.126
Yes	42.19 ± 23.202
Don't know	40.50 ± 26.811
*p*-value	0.869
**Family history of atrial fibrillation**	
No	41.75 ± 26.409
Yes	45.21 ± 23.601
Don't know	38.52 ± 23.388
*p*-value	0.168
**Have you ever had/Do you have stroke? If yes, was this in the last 12 months?**	
No	42.32 ± 25.206
Yes	43.64 ± 19.508
*p*-value	0.856
**Have you ever had/Do you have heart attack ? If yes, was this in the last 12 months?**	
No	41.58 ± 25.200
Yes	52.04 ± 20.937
*p*-value	0.026
**Have you ever had/Do you have Angina? If yes, was this in the last 12 months?**	
No	41.94 ± 25.087
Yes	50.34 ± 23.218
*p*-value	0.140
**Have you ever had/Do you have transient ischaemic attack (TIA)? If yes, was this in the last 12 months?**	
No	42.34 ± 25.076
Yes	42.92 ± 24.714
*p*-value	0.943
**Have you ever had/Do you have Diabetes? If yes, was this in the last 12 months?**	
No	41.98 ± 25.084
Yes	45.15 ± 24.765
*p*-value	0.408
**Have you ever been told by a doctor that you have high blood pressure?**	
No	42.53 ± 25.099
Yes, but without medication	39.09 ± 24.428
Yes, with medication	42.12 ± 25.293
*p*-value	0.891
**Have you ever been told by a doctor that you have high cholesterol?**	
No	41.51 ± 25.118
Yes, but without medication	43.18 ± 27.818
Yes, with medication	49.04 ± 20.066
*p*-value	0.218
**Have you ever been told by a doctor that you have abnormal heart rhythm (atrial fibrillation)?**	
No	40.94 ± 25.060
Yes, but without medication	53.42 ± 20.744
Yes, with medication	57.42 ± 21.592
*p*-value	0.002
**Where do you get most of your health information? Internet**	
No	50.49 ± 28.028
Yes	38.41 ± 22.464
*p*-value	<0.001
**Where do you get most of your health information? Newspaper/Magazine**	
No	41.84 ± 25.201
Yes	45.80 ± 23.858
*p*-value	0.280
**Where do you get most of your health information? Television**	
No	43.57 ± 25.781
Yes	39.05 ± 22.672
*p*-value	0.086
**Where do you get most of your health information? Health/fitness center**	
No	41.89 ± 25.148
Yes	47.60 ± 23.474
*p*-value	0.206
**Where do you get most of your health information? Friends**	
No	44.21 ± 25.691
Yes	36.01 ± 21.609
*p*-value	0.002
**Where do you get most of your health information? Church**	
Yes	42.59 ± 24.971
No	16.12 ± 20.355
*p*-value	0.044
**Where do you get most of your health information? Alternative practitioners**	
No	42.49 ± 25.218
Yes	38.02 ± 19.068
*p*-value	0.419
**Where do you get most of your health information?Nurse**	
No	42.32 ± 25.357
Yes	42.61 ± 22.483
*p*-value	0.938
**Where do you get most of your health information? Doctor**	
No	45.83 ± 26.381
Yes	39.62 ± 23.628
*p*-value	0.014
**Where do you get most of your health information? Family member**	
No	44.79 ± 25.207
Yes	31.92 ± 21.494
*p*-value	<0.001
**Where do you get most of your health information? Don't know**	
No	41.37 ± 24.447
Yes	53.68 ± 29.142
*p*-value	0.025

In the multivariate analysis, the mean STAT scores of participants who get their information from the internet, a family member and church were statistically significantly lower (ß = −10.177, *p* < 0.001; ß = −9.069, *p* = 0.001; and ß = −36.975, *p* = 0.001, respectively). Also, no association was found between the SKT score and the STAT score (Table 4).

**Table 4:** Multivariate analysis taking the STAT score as the dependent variable.

**Variable**	**Unstandardized Beta**	**Standardized Beta**	**p-value**	**Confidence Interval**	
**Model 1: linear regression taking the sociodemographic factors as independent variables**				
Where do you get most of your health information? Internet	−10.177	−0.191	<0.001	−15.675	−4.679
Where do you get most of your health information? Family Member	−9.069	−0.155	0.001	−14.518	−3.619
Where do you get most of your health information? Church	−36.975	−0.153	0.001	−59.587	−14.364
**Model 2: linear regression taking the SKT score as the independent variable**				
SKT score	−0.004	−0.004	0.930	−0.104	0.095

## Discussion

To date, there's a paucity of information regarding knowledge and response to stroke in the Lebanese community. Hence, it is important to assess the stroke awareness level in order to implement preventive measures especially regarding primary stroke prevention, targeting better outcomes, and diminishing morbidity and mortality.

### Knowledge of Stroke

The current research showed that stroke-related knowledge in the Lebanese community is insufficient. Almost half of the respondents demonstrated a poor level of stroke knowledge which is similar to studies conducted in numerous other countries (26–29, 34, 35, 47–49).

Most of the participants (61%) were capable of identifying the pathological mechanism of stroke by answering that it is due to blockage of the blood supply to the brain. This revealed that Lebanese adults had some awareness about the development of the illness. The understanding of the disease development is important for them to be able to distinguish a stroke from a heart attack which tend to be confused by a large number of people (50–53). This result is in accordance with studies conducted in Malaysia (66.7%) (48) and in Ireland (60.3%) (47), however the percentage is higher than studies conducted in India (31%) (54) and a previous study conducted in Lebanon where 44.6% of the participants stated the pathological mechanism of the disease (55). An explanation could be that in the current study, closed-ended questions were used instead of open-ended questions employed in the previously mentioned studies. Also, it is noteworthy to mention that the Arabic translation of stroke is more descriptive and therefore facilitates the understanding of disease development.

Moreover, there was a lack of knowledge about controllable risk factors of stroke which is a finding that is consistent with many previous studies (26–29, 34, 47, 56–64). This finding may provide an indication into the cause for the higher stroke prevalence in Lebanon compared to other developing countries in the middle East.

The well-recognized risk factors in our research were physical inactivity (70.5%), obesity (69.3%), hyperlipidemia (67.3%), and hypertension (52.9%). Similarly, numerous studies have revealed these 4 as being the most identified risk factors of stroke (22, 59, 65). In contrast, in a recent study conducted in Ethiopia among hypertensive patients, hypertension was the least recognized risk factor (3.6%) (28).

Interestingly, although smoking is a well-known stroke risk factor, it was the least perceived risk factor in our study (9.8%). This finding contrasts with many other studies (22, 59, 65) including a previous study conducted in Lebanon where smoking was one of the most recognized risk factors (55). Furthermore, participants underestimated the most pertinent risk factors of stroke-diabetes and atrial fibrillation-despite their high prevalence among respondents in our research. Only a small percentage recognized that they were important stroke risk factors (37.8 and 17.6%, respectively). Numerous research has also shown poor identification of diabetes and atrial fibrillation as risk factors for stroke (22, 57, 65, 66). On the other hand, participants with diabetes were associated with a higher level of stroke-related knowledge which is a result confirmed by several other studies (22, 67).

Living in Beirut was associated with a significantly higher level of stroke-related knowledge than living in Mount Lebanon. This result needs to be confirmed as the number of participants in Mount Lebanon was larger than that of Beirut (197 and 83, respectively).

In addition, research has demonstrated that educated and younger participants have a higher level of stroke-related knowledge (26, 54–59, 68, 69). Our results ad to the literature by confirming this association. It is noteworthy to mention that in Lebanon; the proportion of the population which is literate is higher among the younger adult population which could explain the latter.

Also, our findings revealed that non-smokers have a higher stroke knowledge level. This finding correlates with other studies conducted in Jordan and in Ireland (34, 47). This may be because non-smokers are more conscious about their health which stops them from smoking hence leading to a pursuit of health-related information.

### Response to Stroke

Our results revealed a poor correlation between stroke symptoms and appropriate action. This may be due to insufficient knowledge to effectively recognize stroke symptoms and hence act accordingly, which is in accordance with a former study conducted in Lebanon (55). A similar result was reported in numerous other studies. The findings showed that knowledge of stroke symptoms was significantly associated with the intent to call 112 (24, 50, 70). Moreover, a study conducted in India showed a significant association between poor knowledge of warning signs and delay in hospital arrival (20).

Interestingly, our results showed that 82.4% participants would call 112 when provided with the diagnosis of stroke which is a finding reported in numerous other studies (66, 71–73). However, this result contrasts with Khalil and Lahoud's study where only 57.7% of the respondents would call 112 when stroke was suspected (54).

Moreover, in our study, only 36.8% of participants chose call 112 in response to specific warning signs. A comparable finding was reported in studies conducted in Australia and the US (24, 45).

In addition, our results revealed that the symptoms for which most participants would call 112 are difficulty speaking presented together with sudden arm and face weakness which is a consistent finding with other studies (45, 47). On the other hand, sudden dizziness was one of the symptoms with the lowest intent of respondents to call 112 which is in accordance to another study conducted in Ireland (47).

Furthermore, our findings showed that getting health information from the internet was negatively linked to adequate response to stroke. Tonsaker (74) highlighted that online health information-being difficult to regulate-can be misleading and hence patients might misinterpret it and make important health decisions that conflicts with appropriate medical practices.

It is important to note that there was no significant association between knowledge of stroke and the decision to call an ambulance in our study which is in agreement with a study conducted in Ireland (47). However, this differs from Lackland's et al. study which revealed a decline in stroke mortality in the US that was attributed to the implementation of interventions and programs resulting in better knowledge of stroke risk factors, hence improving stroke practice (38–42).

Also, our findings showed a positive relationship between having a medical history of atrial fibrillation and heart attack which is a finding that differs from a study conducted in Ireland where no association was found with the presence of any diagnosis of cardiovascular diseases (47) and another research in the US where a positive relationship was seen in participants with no prior history of heart attack (45).

### Clinical Implications

Previous studies have shown that the achievement of primary preventive strategies and early treatment succeeding a stroke is strongly linked to the community's knowledge and practices of stroke (25, 35, 38–41, 56, 75). Sufficient stroke knowledge and perception could lead to rapid and accurate stroke identification, therefore timely management. Hence, public knowledge and the right practices regarding stroke are required, with aims to lessen the burden of stroke.

Our results revealed poor stroke-related knowledge and a poor relationship between stroke symptoms and intent to call 112. These results are disturbing bearing in mind the increasing occurrence of stroke in Lebanon. Therefore, these results warrant the need to develop and implement national community awareness campaigns. The latter can be designed through institutions, universities, medical care facilities such as doctor's clinics and pharmacies. The use of various media methods is also fundamental in order to disseminate the information and raise public knowledge. In addition, the development of health promotion agendas is of outmost importance in order to aid people embrace healthier lifestyles and subsequently reduce the risk of stroke. The provision of health education focusing on stroke risk factors, warning signs and appropriate action is crucial in order to reduce stroke incidence, morbidity and mortality.

Additionally, Lebanon's current economic and financial crisis is resulting in poor available infrastructure, facilities and qualified personnel despite ranking 23rd in the world for healthcare efficiency in 2019 (76). This adds to the importance of raising stroke awareness in the general public as the lack of amenities, services and equipment is alarmingly rising in Lebanon today.

### Limitations and Strengths

Our study has faced numerous limitations. First, the sample in our study is not representative of all of the Lebanese people as our respondents filled a google form survey which would exclude nationals with no access or not familiar with technological advances leading to selection bias. Moreover, it is important to mention that the scales used in the current research are not validated in Lebanon yet. In addition, the questionnaire was designed with close-ended questions which can overestimate stroke knowledge of respondents. Furthermore, all of the respondents' characteristics and answers were self-reported, therefore the degree of true answers or confirming participant's statements is not possible in the current study which may have caused information bias. The current research is the first study aimed to assess stroke knowledge and practice in adults 18 years and above residing in all five Lebanese districts. Previous research only targeted older adults living in Beirut (55). In addition, the weighting of the data according to age and gender allowed to adjust the sample representation in our study.

## Conclusion

In conclusion, the survey findings revealed poor stroke-related knowledge in the Lebanese community. Knowledge of stroke risk factors was low, as was awareness of the need to call 112 in response to stroke symptoms. These elements result in delays in seeking medical care after stroke, with consequential repercussions for stroke outcome. Hence, it is essential to develop health education programs in order to raise public stroke awareness in Lebanon. Further studies are needed on a more representative sample of the Lebanese people, with the utilization of both close-ended and open-ended questions in order to confirm these findings.

## Data Availability Statement

The original contributions presented in the study are included in the article/Supplementary Material, further inquiries can be directed to the corresponding author.

## Ethics Statement

The studies involving human participants were reviewed and approved by Lebanese University Board. The patients/participants provided their written informed consent to participate in this study.

## Author Contributions

SS, PS, and HH contributed to conception and design of the study. SS organized the database, performed statistical analysis, and wrote the manuscript. SH and PS reviewed and approved the analysis. All authors contributed to manuscript revision, read, and approved the submitted version.

## Conflict of Interest

The authors declare that the research was conducted in the absence of any commercial or financial relationships that could be construed as a potential conflict of interest.

## Publisher's Note

All claims expressed in this article are solely those of the authors and do not necessarily represent those of their affiliated organizations, or those of the publisher, the editors and the reviewers. Any product that may be evaluated in this article, or claim that may be made by its manufacturer, is not guaranteed or endorsed by the publisher.
