# Supplementary material for: Knowledge and Response to Stroke Among Lebanese Adults: A Population-Based Survey
Source: Front Public Health. 2022 Jun 3;10:891073. doi: 10.3389/fpubh.2022.891073 (PMC9203897; doi:10.3389/fpubh.2022.891073)
Supplement: Supplementary file 1 [file Table_1.DOCX]

**Appendix A**

| **Table 1. Socio-demographic and socio-economic characteristics of participants** | |
| --- | --- |
| **Variable** | **N (%)** |
| **Gender** | |
| Male | 129 (31.5%) |
| Female | 281 (68.5%) |
| **Marital status** | |
| Single | 198 (48.3%) |
| Married | 197 (48%) |
| Divorced | 15 (3.7%) |
| **Age** | |
| 18-44 | 318 (77.6%) |
| 45-64 | 70 (17.1%) |
| 65 and above | 22 (5.4%) |
| **Level of education** | |
| Elementary/complementary education | 9 (2.2%) |
| Secondary education | 35 (8.5%) |
| University education | 365 (89%) |
| **Professional status** | |
| Worker | 257 (62.7%) |
| Unemployed/Retired | 153 (37.3%) |
| **Social security insurance** | |
| No | 156 (38%) |
| Yes | 254 (62%) |
| **Region** | |
| Beirut | 83 (20.2%) |
| Mount Lebanon | 197 (48%) |
| Bekaa | 21 (5.1%) |
| North Lebanon | 93 (22.7%) |
| South Lebanon | 13 (3.2%) |
| Nabatieh | 3 (0.7%) |
| **Current smoking** |  |
| Every day | 76 (18.5%) |
| Occasionally | 97 (23.7%) |
| Never | 237 (57.8%) |
| **Have you ever smoked at least 100 cigarettes in your entire life? (5 packs=100 cigarettes)** | |
| No | 314 (76.6%) |
| Yes | 96 (23.4%) |
| **Daily Alcohol Consumption** | |
| Every day | 2 (0.5%) |
| Occasionally | 229 (55.9%) |
| Never | 179 (43.7%) |
| **Has any member of your family or a close friend had a stroke?** | |
| No | 226 (55.1%) |
| Yes | 184 (44.9%) |
| **Family history of** | |
| \|  \| No \| \| Yes \| \| Don’t know \| \| \| --- \| --- \| --- \| --- \| --- \| --- \| --- \| \| N \| % \| N \| % \| N \| % \| \| Heart failure \| 316 \| 77.1 \| 44 \| 10.7 \| 50 \| 12.2 \| \| Stroke \| 336 \| 82 \| 47 \| 11.5 \| 27 \| 6.6 \| \| Myocardial infarction \| 213 \| 52 \| 160 \| 39 \| 37 \| 9 \| \| Atrial Fibrillation \| 194 \| 47.3 \| 146 \| 35.6 \| 70 \| 17.1 \| | |
| **Have you ever had/Do you have any of the following conditions? If yes, was this in the last 12 months?** | |
| \|  \| No \|  \| Yes in the last 12 month \|  \| Yes Long ago \|  \| \| --- \| --- \| --- \| --- \| --- \| --- \| --- \| \| N \| % \| N \| % \| N \| % \| \| Stroke \| 398 \| 20.6% \| 2 \| 6.1% \| 10 \| 11.6% \| \| Heart Attack \| 381 \| 19.7% \| 5 \| 15.2% \| 24 \| 27.9% \| \| Angina \| 388 \| 20.1% \| 5 \| 15.2% \| 17 \| 19.8% \| \| Transient Ischaemic Attack (TIA) \| 400 \| 20.7% \| 2 \| 6.1% \| 8 \| 9.3% \| \| Diabetes \| 364 \| 18.9% \| 19 \| 57.6% \| 27 \| 31.4% \| | |
| **Have you ever been told by a doctor or nurse that you have one of the following conditions? Are you still taking medicine, tablets or pills for this condition?** | |
| \|  \| Ever diagnosed?  No \| \| Yes but without medication \| \| Yes with medication \| \| \| --- \| --- \| --- \| --- \| --- \| --- \| --- \| \|  \| N \| % \| N \| % \| N \| % \| \| High Blood Pressure \| 357 \| 33.4% \| 12 \| 16.7% \| 40 \| 47.1% \| \| High Cholesterol \| 342 \| 32.0% \| 38 \| 52.8% \| 29 \| 34.1% \| \| Abnormal Heart Rhythm (Atrial fibrillation) \| 370 \| 34.6% \| 22 \| 30.6% \| 16 \| 18.8% \| | |
| **Where do you get most of your health information?** | |
| \|  \| No \| \| Yes \| \| \| --- \| --- \| --- \| --- \| --- \| \|  \| N \| % \| N \| % \| \| Television \| 298 \| 29.7% \| 112 \| 27.3% \| \| Church \| 56 \| 5.6% \| 354 \| 86.3% \| \| Doctor \| 103 \| 10.3% \| 307 \| 74.9% \| \| Nurse \| 36 \| 3.6% \| 374 \| 91.2% \| \| Newspaper/ Magazine \| 97 \| 9.7% \| 313 \| 76.3% \| \| Family Member (I remove it?) \| 4 \| .4% \| 406 \| 99.0% \| \| Friends \| 14 \| 1.4% \| 396 \| 96.6% \| \| Internet \| 53 \| 5.3% \| 357 \| 87.1% \| \| Health/Fitness Center \| 226 \| 22.6% \| 184 \| 44.9% \| \| Alternative Practitioners \| 85 \| 8.5% \| 325 \| 79.3% \| \| Don't know \| 30 \| 3.0% \| 380 \| 92.7% \| | |
